# Gesundheitliche Ungleichheit in einem Gesundheitswesen mit 2 Vollversicherungssystemen? Sekundäranalysen des Mikrozensus bei Versicherten der Gesetzlichen Krankenversicherung und Privaten Krankenversicherung in Deutschland

**DOI:** 10.1007/s00103-025-04142-1

**Published:** 2025-10-10

**Authors:** Alfons Hollederer

**Affiliations:** Fachbereich 01 Humanwissenschaften, Institut für Sozialwesen, Universität Kassel, Arnold-Bode-Str. 10, 34109 Kassel, Deutschland

**Keywords:** Krankenversicherung, Gesundheitswesen, Gesundheitsversorgung, Gesundheitsausgaben, EU-SILC-Survey, Health insurance, Delivery of Health Care, Healthcare, Health expenditure, EU-SILC Survey

## Abstract

**Hintergrund:**

Das Gesundheitswesen in Deutschland ist durch die Parallelität der Gesetzlichen Krankenversicherung (GKV) und der Privaten Krankenversicherung (PKV) geprägt. Diese Konstellation von 2 Vollversicherungssystemen bei gleichzeitiger Krankenversicherungspflicht ist international einmalig. Ziel der Studie ist es, neue Erkenntnisse zur gesundheitlichen Ungleichheit der Versicherten zu gewinnen.

**Methoden:**

Die Sekundärdatenanalyse wertete den repräsentativen Mikrozensus 2021 und 2022 (inkl. Statistik der Europäischen Union über Einkommen und Lebensbedingungen) mittels Korrelations- und Regressionsanalysen aus.

**Ergebnisse:**

Die Vergleichsanalysen zeigen signifikante Unterschiede zwischen PKV und GKV nach soziodemografischen und erwerbsbiografischen Charakteristika und verifizieren eine gesundheitliche Ungleichheit. Logistische Regressionsanalysen ergeben für PKV-Versicherte, dass sie im Vergleich zu GKV-Versicherten eine doppelt so hohe Wahrscheinlichkeit für eine sehr gute oder gute subjektive Gesundheit (OR = 2,09) und fast halbierte Wahrscheinlichkeiten für Gesundheitseinschränkungen (OR = 0,53) und chronische Krankheiten (OR = 0,64) unter Berücksichtigung von soziodemografischen Einflussfaktoren haben. Bei subjektiv ähnlich hohem Versorgungsbedarf konsultierten sie häufiger Fach- sowie Zahnärzte und -ärztinnen, während GKV-Versicherte öfter Hausärzte und -ärztinnen aufsuchten. PKV-Versicherte verhielten sich bezüglich Rauchen, Bewegung und Ernährung gesünder, konsumierten aber stärker Alkohol. Für GKV-Versicherte stellten Gesundheitsausgaben und Zuzahlungen im Vergleich zur PKV häufiger eine „große Belastung“ im Haushalt dar, insbesondere für die Zahnmedizin (10,0 % vs. 4,1 %).

**Diskussion:**

Die Ergebnisse haben Implikationen für die Gesundheitssystemgestaltung und sind relevant für die Prävention und Gesundheitsberichterstattung.

## Abstract (en)

**Introduction:**

The German healthcare system is characterised by the parallelism of private health insurance (PKV) and statutory health insurance (GKV). This constellation of two full insurance systems with simultaneous mandatory health insurance is unique worldwide. This study aims to gain new scientific findings on health inequalities.

**Method:**

The secondary data analysis of the representative 2021 and 2022 microcensus survey (including the European Union Statistics on Income and Living Conditions) uses correlation and regression analyses.

**Results:**

Comparative analyses reveal significant differences in socio-demographic characteristics and employment history between PKV and GKV and verify health inequalities between the two groups. Considering socio-demographic factors, logistic regression analyses show that the probability of very good or good self-rated health is twice as high for those with PKV compared to those with GKV (OR = 2.09). The likelihood of health impairments (OR = 0.53) and chronic diseases (OR = 0.64) is reduced by almost half. Despite having similar subjective needs for healthcare, those with PKV consulted medical specialists and dentists more frequently, while those with GKV consulted family doctors or general practitioners more frequently. People with PKV displayed healthier behaviours than those with GKV with regard to tobacco consumption, exercise and diet. However, they consumed more alcohol. People with GKV were more likely than those with PKV to report health expenditure and out-of-pocket payments as a “major burden”, especially for dental care (10.0% vs. 4.1%).

**Discussion:**

The results have implications for the design of healthcare systems and are relevant for prevention and health reporting.

## Hintergrund

Im internationalen Vergleich [1] fällt das deutsche Gesundheitssystem durch eine einmalige Konstellation auf: Es hält mit der Gesetzlichen Krankenversicherung (GKV) und der Privaten Krankenversicherung (PKV) 2 Vollversicherungssysteme parallel vor und es besteht gleichzeitig allgemeine gesetzliche Krankenversicherungspflicht (im Sinne des § 193 Versicherungsvertragsgesetz, VVG). Diese Parallelität von PKV und GKV wird in der Gesundheitspolitik kontrovers diskutiert und häufig als „2-Klassen-Medizin“ kritisiert [2].

Hinter der deutschen Besonderheit steht ein historisch gewachsenes Mischsystem aus grundlegend verschiedenen Gesundheitssystemmodellen. Neben dem in Deutschland aufgebauten solidarischen Sozialversicherungsmodell („Bismarck-Modell“) wurden Teilsysteme für bestimmte Bevölkerungsgruppen nach dem marktwirtschaftlichen Modell implementiert. Die beiden großen Versicherungssysteme PKV und GKV decken trotzdem nicht vollständig die Bevölkerung ab, da es darüber hinaus noch ein staatliches Fürsorgemodell gibt. Speziell für die Beamtenschaft gibt es mit der „Beihilfe“ Elemente nach dem Versorgungsprinzip sowie für Sondergruppen wie Soldaten und Soldatinnen, Polizei oder Berufsfeuerwehr die „Heilfürsorge“. Daneben werden für Gefangene die „Gesundheitsfürsorge“ gemäß § 56 Strafvollzugsgesetz (StVollzG) und für Asylsuchende eine reduzierte „Gesundheitspflege“ gemäß § 3 Asylbewerberleistungsgesetz (AsylbLG) nach den jeweils geltenden landesvollzugsgesetzlichen Regelungen sichergestellt. Die 3 grundlegenden Gesundheitssystemmodelle entstammen unterschiedlichen Paradigmen und Zeiten. Sie bringen in der heutigen Praxis nicht nur verschiedene Finanzierungsarten (paritätische Beitragsfinanzierung, private Finanzierung und Steuerfinanzierung), sondern auch Trägerschaften, Organisationsformen und Steuerungsmechanismen mit sich [3].

Im Gegensatz zur öffentlich-rechtlichen GKV, die primär über das Sozialgesetzbuch (SGB) V „Gesetzliche Krankenversicherung“ nach dem Solidaritätsprinzip reguliert wird, ist die PKV privatwirtschaftlich organisiert. Die privaten Krankenkassen sind zumeist gewinnorientierte Aktiengesellschaften, aber es gibt darunter auch Versicherungsvereine auf Gegenseitigkeit. Die Beihilfe im Krankheitsfall wird für Beamte und Beamtinnen dagegen über Gesetze und Verordnungen konkretisiert, die jedoch auch nicht ganz einheitlich zwischen den Bundesländern und im Vergleich zum Bund gestaltet sind. Durch die gesetzliche Regelung zur Krankenversicherungspflicht ist nahezu die gesamte Wohnbevölkerung in Deutschland krankenversichert [1, 4]. Die Gesetzliche Krankenversicherung hat 74,5 Mio. Mitglieder [5]. Die Private Krankenversicherung zählt insgesamt 8,7 Mio. Vollversicherte in Deutschland [6]. Beamte und Beamtinnen machen insgesamt über die Hälfte des Versichertenanteils in der privaten Krankheitsvollversicherung aus [6]. Sie stellen damit die größte Versichertengruppe der PKV dar. Die Abrechnung privatärztlicher Leistungen wird über eine eigene „Gebührenordnung für Ärzte“ (GOÄ) geregelt. Die großen Abrechnungsunterschiede zum „einheitlichen Bewertungsmaßstab“ (EBM) der vertragsärztlichen Versorgung in der GKV verstoßen dabei gegen das Prinzip „gleicher Preis für gleiche Leistungen“. Die Systemunterschiede sind indessen in den Sektoren unterschiedlich ausgeprägt [7].

Trotz der Relevanz für das Gesundheitssystem besteht ein Forschungsdefizit. Es mangelt an Vergleichsanalysen zwischen der GKV und PKV in Bezug auf Gesundheit und Krankenversorgung der Versicherten. Die Gesundheitsberichterstattung baut in Deutschland vor allem auf Gesundheitssurveys, Gesundheitsstatistiken sowie Routinedaten von der GKV auf [8]. Die Gesundheitssurveys sind aber für die PKV aufgrund der kleinen Fallzahlen meistens wenig aussagekräftig. Wissenschaftliche Studien belegen signifikante Unterschiede in der Versicherten- und Morbiditätsstruktur zwischen der GKV und PKV [9–12]. Diese wenigen Studien weisen jedoch laut Huber und Mielck [10] teilweise erhebliche methodische Schwächen auf. Hinzu kommt, dass die PKV selten ihre Routinedaten für die Versorgungsforschung frei zur Verfügung stellt. Für die gesamte Beihilfe in Krankheits‑, Pflege- und Geburtsfällen sind eine fehlende Datentransparenz und Versorgungsforschung zu konstatieren.

Weitere offene Forschungsfragen betreffen die Zugänge in die Gesundheitsversorgung in Abhängigkeit von der Krankenversicherungsart. Trotz des hohen Versicherungsgrades in der Bevölkerung stellen sich Fragen nach Zugangsbarrieren. Unterschiedliche Vergütungssysteme zwischen der Gesetzlichen und Privaten Krankenversicherung könnten zu Fehlanreizen im System und zu Versorgungsunterschieden in der Qualität und den Angebotsstrukturen führen. Eine Studie von Sundmacher und Ozegowski [13] zeigte für die Kreise in Deutschland auf, dass höhere Anteile der Privatversicherten in der Bevölkerung mit einem Anstieg der Fach- und Hausarztdichte korrespondierten. In der Gesundheitspolitik und Öffentlichkeit werden unterschiedliche Wartezeiten seit Langem diskutiert. Studien [9, 14, 15] legten offen, dass gesetzlich Krankenversicherte im Mittel länger auf einen Termin in Facharztpraxen warten müssen als privat Versicherte.

Von Interesse sind auch die jeweiligen Versichertenstrukturen in der GKV und PKV sowie die damit verbundenen „Versichertenrisiken“ in gesundheitsökonomischer Hinsicht [16]. Neben Selbstständigen sowie Beamten und Beamtinnen können sich auch Arbeitnehmende bzw. Angestellte mit einem Einkommen oberhalb der Versicherungspflichtgrenze privat versichern. Versicherungsfreiheit besteht für Arbeitnehmende in der GKV, wenn ihr Einkommen die geltende Jahresarbeitsentgeltgrenze gemäß § 6 SGB V überschreitet (2021: 64.350 €). Aufgrund der gesetzlichen Vorgaben unterscheiden sich die Durchschnittseinkommen der Versicherten in der Gesetzlichen und Privaten Krankenversicherung deutlich. Geschlechtsspezifische Aspekte und familiäre Konstellationen sind insbesondere im Zusammenhang mit der beitragsfreien Familienmitversicherung der GKV relevant.

Gleichwohl bilden PKV-Versicherte keine homogene Gruppe, da auch innerhalb der PKV Unterschiede im sozioökonomischen Status festzustellen sind. Die PKV kennt Tarifvielfalt und Wahlfreiheit und hat mittlerweile Basis‑, Standard- und Notlagentarife im Portfolio. Dahinter stehen auch vertragliche Ausschlüsse von bestimmten Leistungen in der Gesundheitsversorgung. Diese Problematik hat vor allem in der Versichertengruppe der Selbstständigen mit den Arbeitsmarktreformen in den 2000er-Jahren und den staatlich unterstützten Existenzgründungsprogrammen aus Arbeitslosigkeit heraus („Ich-AG“) stark zugenommen.

Während sozial bedingte gesundheitliche Ungleichheit in Deutschland bereits im Allgemeinen umfassend untersucht wurde [14, 17, 18], liegen jedoch nur wenige vergleichende Studien zur GKV und PKV im Speziellen vor.

Das Ziel der vorliegenden Arbeit ist es daher, durch Sekundärdatenanalysen des Mikrozensus 2021 und 2022 neue wissenschaftliche Erkenntnisse zur gesundheitlichen Ungleichheit zwischen den Versicherten der GKV und der PKV zu gewinnen. Die Vergleichsanalysen zielen darauf, Gesundheitszustände, Versorgungsbedarf, Inanspruchnahme der Gesundheitsversorgung sowie Belastungen durch Gesundheitsausgaben unter Berücksichtigung von soziodemografischen Merkmalen und erwerbsbiografischen Charakteristika zu explorieren. Die Vergleichsanalysen setzen dabei auf der Ebene der Versicherten an und untersuchen folgende Forschungshypothesen:

1. Aufgrund der rechtlichen Aufnahmebestimmungen in der PKV und der Beihilfeverordnungen wird eine soziale Selektivität erwartet. Daher unterscheiden sich die Versichertenstrukturen zwischen der PKV und GKV nach soziodemografischen Merkmalen und erwerbsbiografischen Charakteristika.
2. Die Versicherten in der PKV sind gesünder als die Versicherten in der GKV gemessen am subjektiven allgemeinen Gesundheitszustand, gesundheitlichen Einschränkungen und chronischen Krankheiten. Die Analysen berücksichtigen soziodemografische Faktoren, die bekanntermaßen Einfluss auf die Zielparameter nehmen und Vergleiche konfundieren können.
3. Die Versicherten in der PKV haben einen geringeren medizinischen Versorgungsbedarf und nehmen weniger ambulante ärztliche Leistungen (Allgemeinmedizin, Zahnmedizin, Facharztrichtungen) als die Versicherten in der GKV in Anspruch.
4. Die Belastungen durch Gesundheitsausgaben und Zuzahlungen sind für PKV-Versicherte geringer als für GKV-Versicherte.
5. Die Gesundheitsverhaltensweisen (Tabakkonsum‑, Alkoholkonsum‑, Bewegungs- und Ernährungsverhalten) sind bei Versicherten in der PKV günstiger als die der Versicherten in der GKV.

## Methoden

Der Mikrozensus bildet die Datenbasis für die amtliche Repräsentativstatistik über die Bevölkerung in Deutschland und wird von den statistischen Ämtern des Bundes und der Länder durchgeführt [19, 20]. Für den Mikrozensus werden Privathaushalte am Hauptwohnsitz nach einem Zufallsverfahren gezogen und alle darin lebenden Personen befragt. Es gibt ein Kernfragenprogramm und wechselnde Schwerpunktthemen. Der Mikrozensus wurde vor Kurzem neu konzipiert [21, 22] und integriert seitdem die jährliche europäische Gemeinschaftsstatistik über Einkommen und Lebensbedingungen (EU-SILC) mit Gesundheitsfragen in einer Unterstichprobe [20]. Der für den EU-SILC im Jahr 2021 realisierte Netto-Stichprobenumfang betrug 40.683 Haushalte und 82.203 Personen [23]. Der Erhebungszeitraum für den EU-SILC war Februar bis August. Die Unit-Non-Response liegt im Mikrozensus 2021 nur bei 17 % [20]. Im EU-SILC 2022 umfasste dann der Netto-Stichprobenumfang 36.661 Haushalte mit 74.080 Personen bei einer Unit-Non-Response von 14 % [19].

Das Hochrechnungsverfahren beim Mikrozensus nahm das Statistische Bundesamt [24] über Kompensationsfaktoren in einer 2‑Phasen-Hochrechnung vor, um die zufallsbedingten und systematischen Fehler der Stichprobe auszugleichen. Die Daten wurden durch dezentrale Befragung mittels Laptop-Interview (CAPI/CATI), Online-Befragung (CAWI) und schriftlicher Befragung gewonnen.

Für den Großteil der Fragen im Mikrozensus besteht Auskunftspflicht [23]. Es sind sogenannte Proxy-Interviews zulässig, daher darf ein Haushaltsmitglied stellvertretend für andere Haushaltsangehörige antworten. Die Mikrozensus-Befragung eruiert umfassend demografische, sozioökonomische und erwerbsbiografische Variablen (Alter, Geschlecht, Erwerbsstatus, Stellung im Beruf, Arbeitslosigkeit, Sozialleistungen etc.; [19, 20]). Der Mikrozensus integriert auch die Arbeitskräfteerhebung (LFS) für den europäischen Vergleich. Die Erhebungskonzeption von Erwerbstätigkeit und Erwerbslosigkeit orientiert sich dabei an dem Labour-Force-Konzept der Internationalen Arbeitsorganisation (ILO; [25]).

In Tab. 1 werden die Erhebungsinstrumente (Fragestellungen und Antwortoptionen) dargestellt, die für die vorliegende Analyse verwendet wurden. Ein zentrales Einzelitem für die vorliegende Arbeit ist die auskunftspflichtige Frage nach dem Krankenversicherungsschutz für das Vorjahr. Hierdurch kann zwischen GKV- und PKV-Versicherten unterschieden werden. Wenn es im Vorjahr einen Wechsel des Versichertenstatus unterjährig gab, wurde für die vorliegende Auswertung anhand der Zahl der berichteten Monate der überwiegende Status kodiert.

Die subjektive allgemeine Gesundheit gilt als geeigneter Proxy-Indikator für den objektiven Gesundheitszustand [26] und wird von der Weltgesundheitsorganisation (WHO) für den Einsatz in derartigen Gesundheitssurveys empfohlen [27].

Ein weiteres Erhebungsinstrument ist der „Global Activity Limitation Indicator“ (GALI; [28]), der mit 3 Fragen die Gesundheitseinschränkungen bei alltäglichen Aktivitäten, die schon mindestens 6 Monate anhalten, eruiert. Der GALI wird von der Europäischen Union für das Monitoring für die Europäische Strategie zugunsten von Menschen mit Behinderungen eingesetzt und konzeptionell als geeigneter Proxy-Indikator für die Messung von Behinderung gewertet [28].

Die Vergleichsanalysen untersuchen darüber hinaus Arbeitsunfähigkeit, medizinischen Versorgungsbedarf, Inanspruchnahme der Gesundheitsversorgung, Belastungen durch Gesundheitsausgaben sowie Gesundheitsverhaltensweisen. Die Items werden in den Tab. 1, 2 und 3 (aus Platzgründen) dargestellt.

**Tab. 1:** Erhebungsinstrumente im Mikrozensus 2021 und 2022

Variable	Fragestellung	Antwortoptionen	Auskunft	Jahr
*Krankenversicherungsschutz*	„In welcher Art waren Sie im Jahr 2020 krankenversichert? Bitte geben Sie bei der entsprechenden Versicherungsart die Anzahl der Monate an, in der das jeweilige Versicherungsverhältnis bestand.“	(1) in einer gesetzlichen Krankenversicherung … a) selbst pflichtversichert; b) selbst freiwillig versichert; c) als Familienangehörige/-r versichert; d) als Student/-in in der Krankenversicherung; e) als Student/-in freiwillig versichert	Pflichtig	Mikrozensus 2021 und 2022
		(2) in einer privaten Krankenversicherung … a) selbst versichert; b) als Familienangehörige/-r versichert; c) als Student/-in versichert		
		(3) Ich hatte Anspruch auf Krankenversorgung im Rahmen der Heilfürsorge		
		(4) Ich war nicht versichert		
*Subjektive allgemeine Gesundheit*	„Wie ist Ihr Gesundheitszustand im Allgemeinen?“	1) sehr gut 2) gut 3) mittelmäßig 4) schlecht 5) sehr schlecht	Freiwillig	Mikrozensus 2021
*Chronische Krankheit*	„Haben Sie eine chronische Krankheit oder ein lang andauerndes gesundheitliches Problem?“ Damit gemeint sind Krankheiten oder gesundheitliche Probleme, die mindestens 6 Monate andauern oder voraussichtlich andauern werden	1) ja 2) nein	Freiwillig	Mikrozensus 2021
*Global Activity Limitation Indicator (GALI)*	A) „Sind Sie dauerhaft durch ein gesundheitliches Problem bei Tätigkeiten des normalen Alltagslebens eingeschränkt?“	1) ja 2) nein	Freiwillig	Mikrozensus 2021
	B) „Wie stark sind Sie bei Tätigkeiten des normalen Alltagslebens eingeschränkt?“	1) stark eingeschränkt 2) mäßig eingeschränkt		
	C) „Wie lange dauert Ihre Einschränkung bereits an?“	1) weniger als 6 Monate 2) 6 Monate oder länger		
*Nichtarbeit*	„Gab es in der Berichtswoche (weitere) Tage, an denen Sie aufgrund von Krankheit, Verletzungen oder vorübergehender Einschränkung nicht gearbeitet haben?“	1) ja 2) nein	Pflichtig	Mikrozensus 2021 und 2022
*Belastung Gesundheitsausgaben*	A) „Denken Sie bitte an die Ausgaben oder Zuzahlungen, die Ihr Haushalt in den letzten 12 Monaten für medizinische Untersuchungen und Behandlungen hatte Nicht gemeint sind die Beiträge zur Krankenversicherung, Ausgaben für zahnärztliche Leistungen oder Kosten für Arzneimittel Welche der folgenden Aussagen für die ärztliche Versorgung trifft zu? Die Kosten für ärztliche Versorgung sind für den Haushalt …“	1) eine große Belastung 2) eine gewisse Belastung 3) keine Belastung 9) trifft nicht zu, kein Bedarf an …	Freiwillig	Mikrozensus 2022
	B) „… für zahnärztliche/kieferorthopädische Untersuchungen und Behandlungen …“			
	C) „… für Arzneimittel (verschreibungspflichtige und nicht-verschreibungspflichtige) …“			
*Ambulante Versorgung*	A) „Wie oft haben Sie in den letzten 12 Monaten einen Zahnarzt, Kieferorthopäden oder einen anderen Zahnpflegeexperten aufgesucht, um sich selbst beraten, untersuchen oder behandeln zu lassen?“	1) gar nicht 2) 1- bis 2‑mal 3) 3- bis 5‑mal 4) 6- bis 9‑mal 5) 10-mal oder mehr	Freiwillig	Mikrozensus 2022
	B) „… einen Hausarzt oder Allgemeinmediziner …“			
	C) „… Facharzt (z. B. Augenarzt, Hautarzt, Orthopäden, Frauenarzt, Physiotherapeuten, Psychotherapeuten) …“			
*Suchtverhalten*	A) „Wie oft haben Sie in den letzten 12 Monaten Alkohol gleich welcher Art getrunken (z. B. Bier, Wein, Sekt, Spirituosen, Schnaps, Cocktails, alkoholische Mischgetränke, Liköre, hausgemachter oder selbstgebrannter Alkohol)?“	1) täglich 2) ein paar Mal in der Woche 3) ein paar Mal im Monat 4) ein paar Mal im Jahr 5) überhaupt nicht	Freiwillig	Mikrozensus 2022
	B) „Wie oft haben Sie in den letzten 12 Monaten Tabakprodukte (z. B. Zigaretten, Pfeifentabak, Wasserpfeife) geraucht? Dazu zählen auch elektronische Zigaretten oder ähnliche elektronische Produkte, z. B. E‑Shisha, E‑Pfeife.“			
*Bewegungs- und Ernährungsverhalten*	A) „Denken Sie an Sport, Fitness und körperliche Aktivität in der Freizeit, z. B. (Nordic‑)Walking, Ballsport, Joggen, Fahrradfahren, Schwimmen, Aerobic, Rudern oder Badminton. Wie oft üben Sie in einer typischen Woche mindestens 10 min ohne Unterbrechung Sport, Fitness oder körperliche Aktivität in der Freizeit aus?“	1) mehrmals täglich 2) einmal täglich 3) 4- bis 6‑mal pro Woche 4) 1- bis 3‑mal pro Woche 5) weniger als einmal pro Woche 6) nie	Freiwillig	Mikrozensus 2022
	B) „Wie oft essen Sie Obst?“			
	C) „Wie oft essen Sie Gemüse oder Salat?“			

Der Zugang zu den für die Wissenschaft frei zur Verfügung gestellten Datensätzen erfolgte über die On-Site-Nutzung des Mikrozensus 2021 (10.21242/12211.2021.00.00.1.1.1; [29]) und 2022 (10.21242/12211.2022.00.00.1.1.0; [30]) an einem Gastwissenschaftsarbeitsplatz in den Forschungsdatenzentren der statistischen Ämter des Bundes und der Länder.

In der Methodik verwendet der Untersuchungsansatz deskriptive Statistik, Korrelationsanalytik und Regressionsanalysen. Zum Vergleich von nominalen Daten zwischen Personengruppen werden Chi-Quadrat-Tests nach Pearson durchgeführt. Das Chi-Quadrat-Verfahren vergleicht die beobachteten Häufigkeitsverteilungen mit den gemäß der Nullhypothese erwarteten Häufigkeiten. Als Zusammenhangsmaße für nominalskalierte Variablen werden bei alternativen Variablen die Phi-Koeffizienten verwendet. Die Regressionsanalyse wird dazu eingesetzt, die unabhängigen Variablen, die die Unterschiede bei der abhängigen Variable hervorrufen, zu identifizieren. Die B‑Regressionskoeffizienten geben an, ob der Zusammenhang zwischen der unabhängigen und abhängigen Variable positiv oder negativ ist. Für die Beurteilung der Einflussgröße wird der Effekt-Koeffizient EXP(B) verwendet. Dieser gibt den Faktor der Vervielfachung der Odds Ratio (des „Chancenverhältnisses“) an. Zu den Odds Ratios werden 95 %-Konfidenzintervalle ermittelt. Die Einschlussgrenze der Kovariaten in den logistischen Regressionsanalysen liegt auf einem Signifikanzniveau innerhalb von *p* < 0,05. Als ein Selektionsverfahren wird die Vorwärts-Selektion mit Wald-Kriterium verwendet. Die Berechnungen wurden mit IBM SPSS-Statistics Version 28 (IBM, Armonk, NY, USA) kalkuliert.

## Ergebnisse

Im Mikrozensus 2021 wurden *N* = 7106 Interviews von Personen erhoben, die angaben, dass sie in der PKV im Jahr 2020 ganzjährig oder überwiegend krankenversichert waren. Sie werden *N* = 62.587 Versicherten der GKV für Vergleichsanalysen gegenübergestellt. Die übrigen Befragten, die entweder unter die staatliche Heilfürsorge fallen oder gar keinen Krankenschutz haben, werden in diesen Vergleichsanalysen nicht berücksichtigt. Alle in den Tab. 2 und 3 und Abb. 2 dargestellten Gruppenunterschiede sind bei den hohen Fallzahlen und der Hochrechnung statistisch signifikant. Auf die Ausweisung der *p*-Werte (*p* < 0,001) wird deshalb dort verzichtet.

**Tab. 2:** Soziodemografie und Erwerbsbiografie im Mikrozensus 2021 (Pflichtangaben). Datenquelle: [29]; eigene Berechnungen

Merkmale	Private Krankenversicherung (PKV)	Gesetzliche Krankenversicherung (GKV)	Gesamt
**I. Soziodemografie**			
*Altersgruppen*			
*(n)*	*(7106)*	*(62.587)*	*(69.693)*
0–17	16,7 %	16,5 %	16,5 %
18–49	30,0 %	39,7 %	38,7 %
50–65	29,6 %	24,0 %	24,6 %
66+	23,7 %	19,8 %	20,2 %
*Geschlecht nach Geburtsregister*^*a*^			
*(n)*	*(7095)*	*(62.518)*	*(69.613)*
Männer	58,2 %	48,3 %	49,3 %
Frauen	41,8 %	51,7 %	50,7 %
*Höchster Bildungsstand (ISCED 2011)*			
*(n)*	*(6183)*	*(54.501)*	*(60.684)*
Primär‑/Sekundärbereich (ISCED 1 bis 4)	42,1 %	76,4 %	72,9 %
Tertiärbereich (ISCED 5 bis 8)	57,9 %	23,6 %	27,1 %
*Staatsangehörigkeit*			
*(n)*	*(7106)*	*(62.587)*	*(69.693)*
Deutsche/-r	95,0 %	86,2 %	87,1 %
Ausländer/-in EU-Staat	2,8 %	5,8 %	5,5 %
Ausländer/-in Nicht-EU	2,2 %	8,0 %	7,4 %
*Ost‑/Westdeutschland*			
*(n)*	*(7106)*	*(62.587)*	*(69.693)*
Ostdeutschland (inkl. Berlin)	12,6 %	20,2 %	19,4 %
Westdeutschland	87,4 %	79,8 %	80,6 %
*Kind bis 14 Jahre im Haushalt*			
*(n)*	*(7106)*	*(62.587)*	*(69.693)*
Ja	31,0 %	32,3 %	32,1 %
Nein	69,0 %	67,7 %	67,9 %
**II. Erwerbsbiografie**			
*Erwerbsstatus*			
*(n)*	*(7106)*	*(62.587)*	*(69.693)*
Erwerbstätige	50,9 %	50,0 %	50,1 %
Erwerbslose/Arbeitsuchende Nichterwerbspersonen	1,0 %	2,7 %	2,6 %
Sonstige Nichterwerbstätige	48,1 %	47,3 %	47,4 %
*Leistungsbezug ALG II, Sozialgeld, Kosten der Unterkunft (Haushalt)*			
*(n)*	*(7106)*	*(62.587)*	*(69.693)*
Ja	1,2 %	8,2 %	7,5 %
Nein	98,8 %	91,8 %	92,5 %
*Stellung im Beruf (gegenwärtige Tätigkeit)*			
*(n)*	*(3575)*	*(31.158)*	*(34.733)*
Selbstständige	30,7 %	6,0 %	8,5 %
Beamte/-Anwärter	35,7 %	0,5 %	4,1 %
Angestellte	26,0 %	70,6 %	66,0 %
Übrige (Arbeiter, Sonstige)	7,5 %	22,9 %	21,3 %
*Öffentlicher Dienst (gegenwärtige Tätigkeit)*			
*(n)*	*(3575)*	*(31.150)*	*(34.725)*
Ja	39,1 %	13,7 %	16,3 %
Nein	60,9 %	86,3 %	83,7 %

Alle *p*-Werte < 0,001 (nicht ausgewiesen); *p*-Werte basieren auf χ 2‑Test nach Pearson bzw. Phi; gewichtete Ergebnisse

^a^Keine Ausweisung von „Diverse“ und „Kein Eintrag im Personenstandsregister“, da zu kleine Fallzahlen gemäß der Mikrozensus-Konvention

Tab. 2, Abschnitt I informiert über Unterschiede zwischen den PKV- und GKV-Versicherten nach soziodemografischen Merkmalen. In der PKV ist der Anteil der älteren Versicherten höher als in der GKV. Die PKV-Versicherten sind zum Befragungszeitpunkt im Mittelwert 47,1 Jahre (SE = 0,01) und damit signifikant älter als die GKV-Versicherten mit durchschnittlich 43,6 Jahren (SE = 0,00). Männer sind in der PKV in der Mehrheit und in der GKV in der Minderheit (58,2 % vs. 48,3 %). Besonders deutlich sind jedoch die Bildungsunterschiede zwischen den Versicherungen. Der Anteil der Menschen mit akademischer Bildung oder weiterführender Berufsbildung (Tertiärbereich) ist in der PKV mit 57,9 % rund 2,5-mal so hoch wie in der GKV mit 23,6 %. In der PKV liegt der Anteil der Versicherten mit deutscher Staatsangehörigkeit höher als in der GKV (95,0 % vs. 86,2 %). Außerdem gibt es regionale Unterschiede zwischen Ost- und Westdeutschland. Die PKV hat einen etwas höheren Versichertenanteil in Westdeutschland als die GKV (87,4 % vs. 79,8 %).

Wie Tab. 2, Abschnitt II zeigt, ist sowohl in der PKV als auch in der GKV rund die Hälfte der Versicherten erwerbstätig. Wichtige Unterschiede bestehen jedoch bei den Erwerbslosen, einschließlich der arbeitsuchenden Nichterwerbspersonen: In der PKV liegt ihr Anteil deutlich unter dem der GKV mit 1,0 % vs. 2,7 %. Große Unterschiede offenbaren sich auch bei der Frage nach öffentlichen Leistungen im Haushalt: 2020 erhielten in der PKV lediglich 1,2 % der Versicherten Arbeitslosengeld II (Hartz IV), Sozialgeld oder Kosten der Unterkunft, während der Anteil in der GKV mit 8,2 % deutlich höher war.

Die Gegenüberstellung in Tab. 2 legt erhebliche Strukturunterschiede zwischen den Krankenversicherungen in der Erwerbspopulation offen. In der PKV gehört rund ein Drittel der Erwerbstätigen zur Beamtenschaft und fast ein weiteres Drittel ist selbstständig. In der GKV zählen dagegen über 2 Drittel der Erwerbstätigen zu den Angestellten. In der PKV sind 39,1 % der Erwerbstätigen im Öffentlichen Dienst tätig, während dieser Anteil in der GKV lediglich 13,7 % beträgt.

**Abb. 1:**
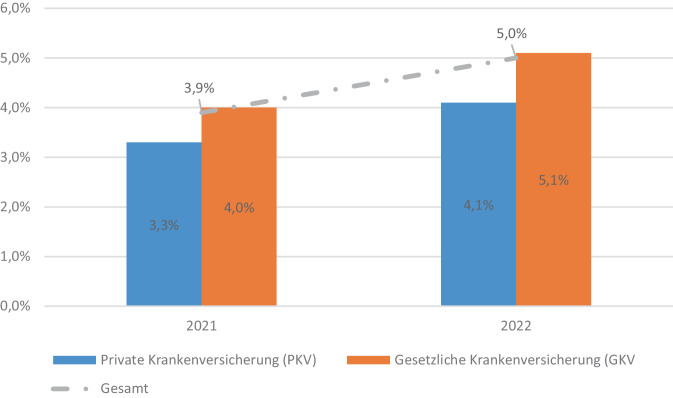
Nichtarbeit in der Berichtswoche aufgrund von Krankheit, Verletzungen oder vorübergehender Einschränkung bei gegenwärtiger Tätigkeit im Mikrozensus 2021 und 2022 (Pflichtangaben). Anmerkung: *n* = 34.733 bzw. *n* = 37.609; Phi (gewichtet) = 0,011; *p* < 0,001 bzw. 0,014; *p* < 0,001. Datenquellen: [29, 30]; eigene Berechnungen

Wie Abb. 1 zeigt, unterscheiden sich die GKV- und PKV-Versicherten in Hinblick auf Arbeitsunfähigkeit signifikant. 3,3 % der Erwerbstätigen in der PKV gaben im Mikrozensus 2021 an, dass sie in der Berichtswoche aufgrund von Krankheit, Verletzungen oder vorübergehender Einschränkung einen oder mehrere Tage nicht gearbeitet haben. In der GKV lag dieser Anteil mit 4,0 % signifikant höher. Diese Pflichtfrage wurde im Mikrozensus 2022 nochmals identisch gestellt. Wie die Abb. 1 demonstriert, nahm die Nichtarbeit wegen Krankheit zwischen den Jahren 2021 und 2022 stark zu, aber die Unterschiede zwischen PKV und GKV blieben mit 4,1 % vs. 5,1 %. Der massive Anstieg steht in Übereinstimmung mit Fehlzeitenreports der Krankenkassen und war demnach vor allem Atemwegserkrankungen und psychischen Erkrankungen im Zuge der COVID-19-Pandemie geschuldet [31].

Tab. 3, Teil I weist die Selbstangaben zu Gesundheit, Krankheit und Behinderung im freiwilligen EU-SILC-Fragenteil des Mikrozensus 2021 aus. In der PKV bewerteten 75,2 % der Versicherten ihren Gesundheitszustand im Allgemeinen als sehr gut oder gut, während dieser Prozentsatz unter den GKV-Versicherten mit 61,5 % signifikant niedriger lag – obwohl sie im Durchschnitt jünger waren. In der PKV sind prozentual auch weniger Menschen mit chronischen Krankheiten und lang andauernden Gesundheitsproblemen als in der GKV versichert (34,4 % vs. 42,3 %).

**Tab. 3:** Gesundheit nach Krankenversicherung im Mikrozensus 2021 und 2022 (EU-SILC-Fragenteil). Datenquelle: [29, 30]; eigene Berechnungen

Merkmale	Private Krankenversicherung (PKV)	Gesetzliche Krankenversicherung (GKV)	Gesamt
**I. Gesundheit, Krankheit und Behinderung im Mikrozensus 2021**			
*Allgemeiner Gesundheitszustand*			
*(n)*	*(3027)*	*(27.415)*	*(30.442)*
Sehr gut	27,5 %	18,9 %	19,7 %
Gut	47,7 %	42,6 %	43,2 %
Mittelmäßig	17,7 %	25,5 %	24,7 %
Schlecht oder sehr schlecht	7,1 %	13,0 %	12,4 %
*Chronische Krankheit bzw. lang andauerndes gesundheitliches Problem*			
*(n)*	*(2887)*	*(26.370)*	*(29.257)*
Ja	34,4 %	42,3 %	41,5 %
Nein	65,6 %	57,7 %	58,5 %
*Global Activity Limitation Indicator (GALI)*			
*(n)*	*(2819)*	*(25.462)*	*(28.281)*
Nicht eingeschränkt	83,9 %	74,5 %	75,4 %
Mäßig eingeschränkt	9,6 %	14,2 %	13,8 %
Stark eingeschränkt	6,5 %	11,3 %	10,8 %
**II. Medizinischer Versorgungsbedarf im Mikrozensus 2021**			
*Zahnärztliche/kieferorthopädische Untersuchung/Behandlung benötigt (letzte 12 Monate)*			
*(n)*	*(2750)*	*(24.619)*	*(27.369)*
Ja	44,7 %	42,8 %	43,0 %
Nein	55,3 %	57,2 %	57,0 %
*Andere ärztliche Untersuchung/Behandlung benötigt (letzte 12 Monate)*			
*(n)*	*(2680)*	*(23.876)*	*(26.556)*
Ja	41,0 %	41,1 %	41,1 %
Nein	59,0 %	58,9 %	58,9 %
**III. Inanspruchnahme der ambulanten Gesundheitsversorgung im Mikrozensus 2022**			
*Zahnarzt, Kieferorthopäden oder Zahnpflegeexperten in den letzten 12 Monaten*			
*(n)*	*(3494)*	*(28.037)*	*(31.531)*
Gar nicht	22,0 %	24,5 %	24,2 %
1- bis 2‑mal	62,0 %	61,3 %	61,4 %
3- bis 5‑mal	12,8 %	11,3 %	11,4 %
6‑mal oder mehr	3,2 %	2,9 %	3,0 %
*Hausarzt oder Allgemeinmediziner in den letzten 12 Monaten*			
*(n)*	*(3495)*	*(28.242)*	*(31.737)*
Gar nicht	19,4 %	14,4 %	15,0 %
1- bis 2‑mal	43,4 %	39,8 %	40,2 %
3- bis 5‑mal	25,3 %	29,1 %	28,7 %
6- bis 9‑mal	6,2 %	8,9 %	8,6 %
10-mal oder mehr	5,7 %	7,8 %	7,5 %
*Facharzt in den letzten 12 Monaten*			
*(n)*	*(3469)*	*(27.812)*	*(31.281)*
Gar nicht	29,9 %	32,3 %	32,0 %
1- bis 2‑mal	36,8 %	36,7 %	36,7 %
3- bis 5‑mal	18,6 %	17,7 %	17,8 %
6- bis 9‑mal	6,4 %	5,9 %	5,9 %
10-mal oder mehr	8,4 %	7,5 %	7,6 %
IV. Gesundheitsverhaltensweisen im Mikrozensus 2022			
*Alkoholkonsum in den letzten 12 Monaten*			
*(n)*	*(3199)*	*(25.544)*	*(28.743)*
Täglich	7,7 %	4,5 %	4,8 %
Ein paar Mal in der Woche	24,8 %	16,6 %	17,5 %
Ein paar Mal im Monat	31,0 %	26,7 %	27,2 %
Ein paar Mal im Jahr	19,4 %	22,4 %	22,1 %
Überhaupt nicht	17,1 %	29,8 %	28,4 %
*Tabakprodukte rauchen in den letzten 12 Monaten*			
*(n)*	*(3307)*	*(26.077)*	*(29.384)*
Täglich	9,7 %	19,0 %	18,0 %
Ein paar Mal in der Woche	2,4 %	3,4 %	3,3 %
Ein paar Mal im Monat	2,3 %	2,3 %	2,3 %
Ein paar Mal im Jahr	2,8 %	2,8 %	2,8 %
Überhaupt nicht	82,8 %	72,6 %	73,7 %
*Sport, Fitness und körperliche Aktivität in der Freizeit*			
*(n)*	*(3309)*	*(26.317)*	*(29.626)*
Mehrmals täglich	22,6 %	19,6 %	20,0 %
Einmal täglich	40,8 %	38,0 %	38,3 %
4- bis 6‑mal pro Woche	13,6 %	13,3 %	13,3 %
1- bis 3‑mal pro Woche	16,4 %	19,6 %	19,3 %
Weniger als einmal pro Woche oder nie	6,6 %	9,5 %	9,2 %
*Obstverzehr*			
*(n)*	*(3309)*	*(26.317)*	*(29.626)*
Mehrmals täglich	22,6 %	19,6 %	20,0 %
Einmal täglich	40,8 %	38,0 %	38,3 %
4- bis 6‑mal pro Woche	13,6 %	13,3 %	13,3 %
1- bis 3‑mal pro Woche	16,4 %	19,6 %	19,3 %
Weniger als einmal pro Woche oder nie	6,6 %	9,5 %	9,2 %
*Gemüse oder Salat essen*			
*(n)*	*(3295)*	*(26.233)*	*(29.528)*
Mehrmals täglich	17,6 %	15,2 %	15,5 %
Einmal täglich	41,3 %	38,0 %	38,3 %
4- bis 6‑mal pro Woche	21,8 %	20,1 %	20,3 %
1- bis 3‑mal pro Woche	16,9 %	21,5 %	21,0 %
Weniger als einmal pro Woche oder nie	2,5 %	5,2 %	4,9 %

Alle *p*-Werte < 0,001 (nicht ausgewiesen); *p*-Werte basieren auf χ 2‑Test nach Pearson bzw. Phi; gewichtete Ergebnisse

Entsprechend dem GALI-Indikator gaben 9,6 % der befragten PKV-Versicherten an, aufgrund ihres lang andauernden gesundheitlichen Problems bei alltäglichen Aktivitäten mäßig eingeschränkt zu sein. Weitere 6,5 % berichteten starke Einschränkungen. Wie Tab. 3, Abschnitt I belegt, sind Versicherte in der GKV wesentlich häufiger von derartigen Gesundheitseinschränkungen mit analogen Anteilen von 14,2 % und 11,3 % betroffen.

Unerwarteterweise wird der subjektive medizinische Versorgungsbedarf in den letzten 12 Monaten von den Versicherten in der PKV und GKV ähnlich hoch eingeschätzt (Tab. 3, Abschnitt II).

Von großem Interesse ist die Inanspruchnahme der ambulanten Gesundheitsversorgung, die aber nur im Mikrozensus 2022 erhoben wurde. Wie die Tab. 3, Abschnitt III im Detail aufzeigt, haben die PKV-Versicherten im Durchschnitt etwas häufiger als die GKV-Versicherten a) Zahnärzte und -ärztinnen, Kieferorthopäden und -orthopädinnen oder andere Zahnpflegeexperten und -expertinnen sowie b) Fachärzte und -ärztinnen in den letzten 12 Monaten aufgesucht, um sich selbst beraten, untersuchen oder behandeln zu lassen. Umgekehrt liegt die durchschnittliche Anzahl der Besuche bei Hausärzten und Hausärztinnen bzw. Allgemeinmediziner(innen) bei den GKV-Versicherten höher.

Der Mikrozensus 2022 eruiert auch die Gesundheitsverhaltensweisen, darunter erstmalig den Alkoholkonsum. Wie Tab. 3, Abschnitt IV informiert, wird überraschenderweise von Versicherten der PKV wesentlich mehr Alkohol als von Versicherten der GKV konsumiert. Es sagten prozentual weniger Versicherte der PKV im Vergleich zur GKV, dass sie überhaupt keinen Alkohol in den letzten 12 Monaten getrunken haben (17,1 % vs. 29,8 %). Die geringeren Abstinenzraten können aber nicht nur auf den höheren Männeranteil (mit stärkerem Alkoholkonsum) in der PKV zurückgeführt werden, denn auch die Frauen in der PKV berichteten mehr Alkoholkonsum als die Frauen in der GKV.

Dagegen liegen die günstigeren Tabak‑, Bewegungs- und Ernährungsverhaltensweisen bei PKV-Versicherten im Erwartungshorizont (Tab. 3, Abschnitt IV). So gaben 9,7 % der PKV-Versicherten an, in den letzten 12 Monaten täglich Tabakprodukte geraucht zu haben, während dieser Prozentsatz in der GKV mit 19,0 % fast doppelt so hoch war. PKV-Versicherte übten auch etwas häufiger als GKV-Versicherte Sport, Fitness oder andere körperliche Aktivitäten in der Freizeit aus. Außerdem ernährten sich PKV-Versicherte gesünder als GKV-Versicherte und konsumierten etwas häufiger Obst, Gemüse und Salat.

**Abb. 2:**
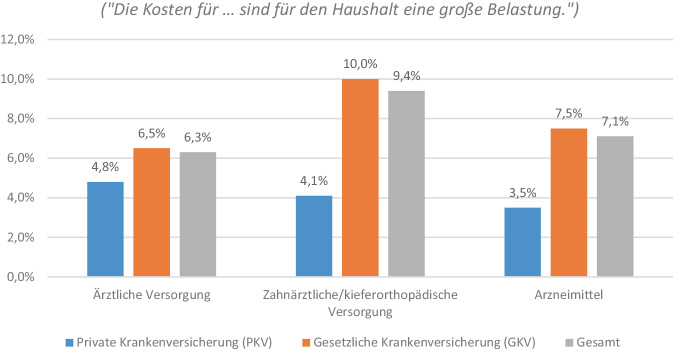
Belastung des Haushalts durch Gesundheitsausgaben und Zuzahlungen in den letzten 12 Monaten im Mikrozensus 2022 (freiwillige Angabe). Anmerkung 1: „eine große Belastung“ versus „eine gewisse Belastung/keine Belastung/trifft nicht zu, kein Bedarf“. Nicht gemeint sind Beiträge zur Krankenversicherung (laut Ausfüllhinweis). Anmerkung 2: Alle *p*-Werte < 0,001 (nicht ausgewiesen); *p*-Werte basieren auf χ 2‑Test nach Pearson; *n* = 36.816. Datenquelle: [30] eigene Berechnungen

Abb. 2 präsentiert die wahrgenommenen Belastungen der Haushalte durch Gesundheitsausgaben und Zuzahlungen in den letzten 12 Monaten im Mikrozensus 2022 (ohne Krankenversicherungsbeiträge). Es klagten mehr als doppelt so viele GKV-Versicherte wie PKV-Versicherte, dass die Ausgaben und Zuzahlungen für zahnärztliche/kieferorthopädische Untersuchungen und Behandlungen (10,0 % vs. 4,1 %) sowie für Arzneimittel eine „große Belastung“ darstellen (7,5 % vs. 3,5 %). Bei den Kosten für die weitere ärztliche Versorgung war die Ungleichheit etwas kleiner (6,5 % vs. 4,8 %).

In Tab. 4 zielen binäre logistische Regressionsanalysen auf a) einen sehr guten oder guten Gesundheitszustand, b) mäßige oder starke Gesundheitseinschränkungen im Alltag und c) das Vorhandensein von chronischen Krankheiten, um vulnerable Gruppen anhand der Odds Ratios zu identifizieren. Sie betrachten jeweils in einem ersten Modell PKV-Versicherte in Relation zu GKV-Versicherten alters- und geschlechtsadjustiert. In einem zweiten Modell beziehen sie jeweils als zusätzliche Kovariaten Regionalbezug, höchsten Bildungsstand, Staatsangehörigkeit und Erwerbsstatus ein. Alle Kovariaten nehmen einen statistisch signifikanten Einfluss auf die Zielparameter in diesen Modellen.

**Tab. 4:** Binäre logistische Regressionsmodelle bei Versicherten der Privaten Krankenversicherung (PKV) und Gesetzlichen Krankenversicherung (GKV) mit Gesundheit als abhängige Variable im Mikrozensus 2021. Datenquelle: [29]; eigene Berechnungen

		Allgemeiner Gesundheitszustand^a^		Global Activity Limitation Indicator (GALI)^b^		Chronische Krankheit/lang andauerndes Gesundheitsproblem^c^	
		Modell 1	Modell 2	Modell 1	Modell 2	Modell 1	Modell 2
		OR	OR	OR	OR	OR	OR
Krankenversicherung	GKV	Ref.	Ref.	Ref.	Ref.	Ref.	Ref.
	PKV	2,51 (2,50–2,52)*	2,09 (2,08–2,09)*	0,46 (0,46–0,46)*	0,53(0,53–0,54)*	0,61 (0,61–0,61)*	0,64 (0,64–0,64)*
Alter	In Jahren	0,95 (0,95–0,95)*	0,96 (0,96–0,96)*	1,05 (1,05–1,05)*	1,03 (1,03–1,03)*	1,04 (1,04–1,04)*	1,03 (1,03–1,03)*
Geschlecht	Frauen	Ref.	Ref.	Ref.	Ref.	Ref.	Ref.
	Männer	1,04 (1,04–1,04)*	0,96 (0,96–0,96)*	0,97 (0,97–0,97)*	1,07 (1,06–1,07)*	0,91 (0,91–0,91)*	0,96 (0,96–0,96)*
Region	Ostdeutschland	Ref.	Ref.	Ref.	Ref.	Ref.	Ref.
	Westdeutschland	–	1,15 (1,14–1,15)*	–	0,89 (0,89–0,89)*	–	0,86 (0,86–0,86)*
Höchste Bildung	Tertiärbereich	Ref.	Ref.	Ref.	Ref.	Ref.	Ref.
	Primär‑/Sekundärbereich	–	0,56 (0,55–0,56)*	–	1,57 (1,57–1,58)*	–	1,24 (1,24–1,24)*
Staatsangehörigkeit	Ausländisch	Ref.	Ref.	Ref.	Ref.	Ref.	Ref.
	Deutsch	–	0,89 (0,89–0,90)*	–	1,24 (1,24–1,25)*	–	1,57 (1,56–1,57)*
Erwerbsstatus	Übrige Personen	Ref.	Ref.	Ref.	Ref.	Ref.	Ref.
	Erwerbstätige	–	2,04 (2,03–2,04)*	–	0,38 (0,38–0,38)*	–	0,59 (0,59–0,59)*
Konstante		3,400668371	2,997832898	−3,810466427	−3,027660706	−2,391413954	−2,192591563
−2 Log-Likelihood		39012762,63	37831630,30	31806321,70	30667293,14	41401230,57	40785253,57
Pseudo‑R^2^ (Nagelkerke)		0,25	0,29	0,19	0,23	0,16	0,18
Pseudo‑R^2^ (Cox & Snell)		0,18	0,21	0,13	0,16	0,12	0,13
*n*		30.410	30.392	28.252	28.235	29.226	29.209

* Signifikanzniveau (Chi-Quadrat-Test nach Pearson) *p* < 0,001. *OR* Odds Ratios (95 %-Konfidenzintervall in Klammern). *Ref*. Referenzkategorie

^a^ 0 = mittelmäßig, schlecht oder sehr schlecht vs. 1 = sehr gut oder gut

^b^ 0 = nicht eingeschränkt vs. 1 = mäßig oder stark eingeschränkt

^c^ 0 = nein vs. 1 = ja

Die Wahrscheinlichkeit von PKV-Versicherten für einen sehr guten oder guten subjektiven Gesundheitszustand ist im Verhältnis zu GKV-Versicherten um den Faktor 2,51 im Modell 1 erhöht. Dieser Faktor erniedrigt sich in der Querschnittsbetrachtung mit allen Kovariaten auf 2,09 im Modell 2. Die Wahrscheinlichkeit für eine positive subjektive Gesundheit von PKV-Versicherten ist demnach in Vergleich zu GKV-Versicherten doppelt so hoch. Dazu konsistent lässt sich diese gesundheitliche Ungleichheit zwischen PKV und GKV auch in den Modellen zu Gesundheitseinschränkungen (GALI) und chronischen Krankheiten nachweisen. Die Odds Ratios sind für PKV-Versicherte unter Adjustierung aller genannten soziodemografischen Kovariaten fast halbiert in Relation zur GKV (OR = 0,53 und OR = 0,64).

## Diskussion

### Limitationen

Als Limitation der Ergebnisse ist vorab darauf hinzuweisen, dass der Krankenversicherungsschutz zwar als Pflichtfrage, aber nur in einem disjunkten Untersample erhoben wurde [32]. Das schränkt die Tiefe der Auswertungsmöglichkeiten aufgrund der Fallzahlen und das Variablenspektrum stark ein. Es ist zu beachten, dass repräsentative Stichprobenerhebungen grundsätzlich mit Zufallsfehlern behaftet sind. Nach dem Qualitätsbericht des Statistischen Bundesamts [20] wurde die Größenordnung des Stichprobenfehlers aber als relativ klein eingeschätzt und dazu viele qualitätssichernde Maßnahmen getroffen.

Die obigen Ergebnisse weisen eine gewisse Unschärfe auf, da der Mikrozensus den Krankenversicherungsschutz des Vorjahrs und nicht zum Berichtszeitpunkt abfragt. Dennoch handelt es sich um einen relativ zeitstabilen Parameter, da beispielsweise der Wechsel aus der PKV zurück in die GKV in den meisten Fällen gesetzlich ausgeschlossen ist. Außerdem wird bei einem Großteil der Gesundheitsitems ebenfalls ein längerer rückwärtiger Zeitraum von 12 Monaten betrachtet.

Die Beantwortung der Gesundheitsfragen im EU-SILC-Fragenteil ist freiwillig. Es wird davon ausgegangen, dass ein etwaiger Item-Nonresponse-Bias ebenso wie eine Antworttendenz in Richtung der sozialen Erwünschtheit die PKV- und GKV-Versicherten gleichermaßen betreffen und deshalb bei den Gruppenvergleichen nur die Randverteilungen tangiert wären. Außerdem erlauben Querschnittsstudien methodisch nur die Bewertung von Assoziationen und nicht Ursache-Wirkungs-Zusammenhänge.

### Erkenntnisse zur gesundheitlichen Ungleichheit zwischen GKV- und PKV-Versicherten

Die Sekundärdatenanalyse des Mikrozensus 2021 und 2022 zeigt

1. gravierende soziodemografische Unterschiede (Alter, Geschlecht, Nationalität, Bildung usw.) sowie gesundheitsbezogene Disparitäten zwischen der GKV und der PKV auf.

Als Hauptergebnis ist in der Gesundheitsperspektive festzuhalten, dass

2. die Gesundheit von PKV-Versicherten weit häufiger als bei GKV-Versicherten als sehr gut oder gut eingeschätzt wurde. Gesundheitliche Ungleichheit wurde beim allgemeinen Gesundheitszustand, bei Behinderung und chronischen Krankheiten nicht nur bivariat, sondern auch multivariat unter Berücksichtigung der soziodemografischen Einflussfaktoren nachgewiesen. Die multivariaten Modelle kontrollieren eine Reihe von potenziellen Selektionseffekten in der Querschnittsbetrachtung. Die Ergebnisse stehen in großer Übereinstimmung mit früheren Studien und anderen Datenquellen [10, 12]. Dennoch besteht weiterer Forschungsbedarf, da die grundlegenden Fragen der Selektion und Kausalität der Gesundheitsunterschiede zwischen PKV und GKV noch nicht geklärt sind und die Studienlage uneinheitlich ist [33–36].
3. Die Inanspruchnahme des ambulanten Gesundheitswesens unterscheidet sich zwischen PKV und GKV. Bei einem ähnlich hoch eingeschätzten medizinischen Versorgungsbedarf in PKV wie GKV konsultierten die PKV-Versicherten etwas häufiger Fachärzte und Zahnärzte; dagegen suchten die GKV-Versicherten häufiger Hausärzte bzw. Allgemeinmediziner in den letzten 12 Monaten auf.
4. Erwartungsgemäß stellen Gesundheitsausgaben und Zuzahlungen für GKV-Versicherte häufiger als für PKV-Versicherte eine „große Belastung“ dar. Das trifft insbesondere auf die Zahnmedizin zu, deren Kosten in Deutschland nach einem OECD-Gesundheitsbericht [1] nur im Umfang von 2 Dritteln durch die gesetzliche Krankenversicherung finanziell abgesichert wird. Gesundheitspolitisch läge bei der Verringerung der Zuzahlungen ein Handlungsansatz zum Abbau der sozial bedingten gesundheitlichen Ungleichheit in Deutschland.
5. Die Ergebnisse zum Gesundheitsverhalten offenbaren insgesamt ein hohes Niveau an vorhandenen Risikofaktoren für die Gesundheit, bei denen Deutschland im europäischen Vergleich besonders schlecht abschneidet [37]. Darunter verhalten sich PKV-Versicherte beim Tabakkonsum, Bewegung und Ernährung im Durchschnitt gesünder als GKV-Versicherte. Eine Ausnahme stellt der starke Alkoholkonsum bei PKV-Versicherten dar. Das ist von Fachinteresse, weil sich die PKV zum einen wenig in der Suchtprävention engagiert [38] und zum anderen in vielen Krankenversicherungstarifen die Kostenübernahme für Krankentagegeld wegen Entziehungskuren/-maßnahmen sowie alkoholbedingten Krankheiten und Unfallfolgen als Leistung ausschließt [39]. In einer konzertierten Verhaltens- und Verhältnisprävention läge daher großes Potenzial für Deutschland.

### Implikationen für die Gestaltung des Gesundheitssystems

Die Vergleichsanalysen sind relevant für die Gesundheitspolitik und Gesundheitssystemgestaltung, denn die GKV und PKV entspringen verschiedenen Modellen. Den Krankenversicherungssystemen sind jeweils eigene Gestaltungsprinzipien, Organisationsformen und Finanzströme immanent. Aus den Ergebnissen des Mikrozensus können Implikationen für die Weiterentwicklung abgeleitet werden, z. B. hinsichtlich der Diskussion um eine Bürgerversicherung [2], die in Tabellen und Abbildungen annähernd dem „Gesamt“ entsprechen würde. Die wesentlich höheren Prävalenzraten an Versicherten mit selbstberichteten gesundheitlichen Einschränkungen (GALI) und chronischen Krankheiten in der GKV deuten bei gleichzeitig niedrigerem Durchschnittsalter auf starke Selektionseffekte im Zugang bei der PKV durch die obligatorischen Gesundheitsprüfungen mit der Möglichkeit der Ablehnung oder von zu hohen individuellen Risikozuschlägen hin. Damit werden das Solidaritätsprinzip und der Wettbewerb an der Systemgrenze zwischen den beiden Vollversicherungen unterlaufen und sie kollidieren. Diese Risikoselektion im Zugang zur PKV wird zulasten der GKV gehen und sich über die höheren Krankheitskosten auf die Finanzierbarkeit des GKV-Systems negativ auswirken.

Die Frage des Krankenversicherungsschutzes von Beamten und Beamtinnen ist für die Gesundheitssystemgestaltung von großer Bedeutung [40]. Das heutige System der anteiligen Beihilfe ist jedoch geprägt von Doppelstrukturen und mangelnder Wahlfreiheit. Beamte und Beamtinnen mit Anspruch auf eine nur anteilige Beihilfe haben angesichts der Kosten zumeist keine echte Wahlfreiheit, weil die GKV prinzipiell nur eine Vollmitgliedschaft kennt und nicht lediglich die Versorgungslücke wie die PKV abdeckt. Laut einer Studie der Bertelsmann-Stiftung [41] würde eine GKV-Versicherung der Beamten und Beamtinnen den Bund und die Länder entlasten, die GKV stärken und sich für die meisten Beamtenhaushalte rechnen. Eine pragmatische Alternative dazu ist, bei der Wahl für die Gesetzliche Krankenversicherung eine „pauschale Beihilfe“ nach dem Vorbild des Landes Hamburg (§ 80 Abs. 2 Hamburgisches Beamtengesetz) zu gewähren. Diesem Modell hat sich mittlerweile die Mehrheit der Bundesländer, aber noch nicht der Bund angeschlossen [42]. In der gesundheitspolitischen Diskussion wird außer Acht gelassen, dass die beihilfeberechtigten privatkrankenversicherten Beamten und Beamtinnen bei PKV und Beihilfe parallel abrechnen müssen und dies doppelte Verwaltungsstrukturen erfordert.

## Fazit

Trotz der hohen Relevanz für die Gesundheitssystemgestaltung gibt es bislang wenige vergleichende Analysen zwischen PKV und GKV in Deutschland. Die vorliegende Sekundärdatenanalyse hat den Mikrozensus und den neu integrierten EU-SILC-Survey als repräsentative Datenquelle für diese Gesundheitsfragen erschlossen und neue wichtige Ergebnisse zur gesundheitlichen Ungleichheit in Deutschland gewonnen. Es gibt jedoch nicht nur gravierende Unterschiede bei der Gesundheit zwischen den Versicherten der PKV und GKV, sondern auch in der Inanspruchnahme der Gesundheitsversorgung. Ein differenzierter Blick lohnt sich bei den heterogenen Ergebnissen zum Gesundheitsverhalten für die Ableitung von Präventionsstrategien. Prävention ist eben nicht nur „Privatsache“! Abschließend ist darauf hinzuweisen, dass die generierten Gesundheitsstatistiken sowohl für die Gesundheitspolitik als auch für die Gesundheitsberichterstattung in Deutschland von Interesse sind. Sie verbessern die unzureichende Datenlage zur Gesundheit und Gesundheitsversorgung insbesondere von den Versicherten der PKV sowie den Beihilfeberechtigten. Bei den Gesundheitsfragen des EU-SILC können die Ergebnisse durch die Wiederholungsbefragungen national fortgeschrieben sowie mit anderen Ländern der EU verglichen werden. Der Ausgestaltung des Krankenversicherungsschutzes von Beamten und Beamtinnen kommt systemanalytisch eine Schlüsselfunktion zu. Eine Bürgerversicherung würde viele Friktionen im Gesundheitssystem überwinden und zu mehr Solidarität führen.

## Data Availability

Die 2 genutzten Scientific Use Files des Mikrozensus 2021 (10.21242/12211.2021.00.00.1.1.1; [29]) und 2022 (10.21242/12211.2022.00.00.1.1.0; [30]) stehen in den Forschungsdatenzentren der statistischen Ämter des Bundes und der Länder für die Wissenschaft nach § 16 Abs. 6 BStatG zur Verfügung. Voraussetzung für die Nutzung des Gastwissenschaftsarbeitsplatzes (GWAP) ist eine Verpflichtung auf die statistische Geheimhaltung nach § 16 Abs. 7 BStatG. Mit dem Ende der Projektlaufzeit werden vertragsgemäß die Duplikate des Mikrozensus sowie die Syntax- und Ausgabedateien am Gastwissenschaftsarbeitsplatz (GWAP) gelöscht.
